# 2-(2-Fluoro­phen­yl)-5-iodo-7-methyl-3-methyl­sulfinyl-1-benzo­furan

**DOI:** 10.1107/S1600536814010459

**Published:** 2014-05-17

**Authors:** Hong Dae Choi, Uk Lee

**Affiliations:** a Department of Chemistry, Dongeui University, San 24 Kaya-dong, Busanjin-gu, Busan 614-714, Republic of Korea; b Department of Chemistry, Pukyong National University, 599-1 Daeyeon 3-dong, Nam-gu, Busan 608-737, Republic of Korea

## Abstract

In the title compound, C_16_H_12_FIO_2_S, the dihedral angle between the plane of the benzo­furan ring system (r.m.s. deviation = 0.023 Å) and that of the 2-fluoro­phenyl ring is 39.78 (7)°. In the crystal, mol­ecules are linked *via* pairs of I⋯π contacts [3.812 (2) Å] and a π–π inter­action between the benzene rings of neighbouring mol­ecules [centroid–centroid distance = 3.821 (2) Å] into inversion dimers. These dimers are further linked by π–π inter­actions between the furan and benzene rings of neighbouring mol­ecules [centroid–centroid distance = 3.668 (2) Å]. The mol­ecules stack along the *a*-axis direction. In addition, C—H⋯O hydrogen bonds are observed between inversion-related dimers.

## Related literature   

For background information and the crystal structures of related compounds, see: Choi *et al.* (2010 ▶, 2012 ▶, 2014 ▶).
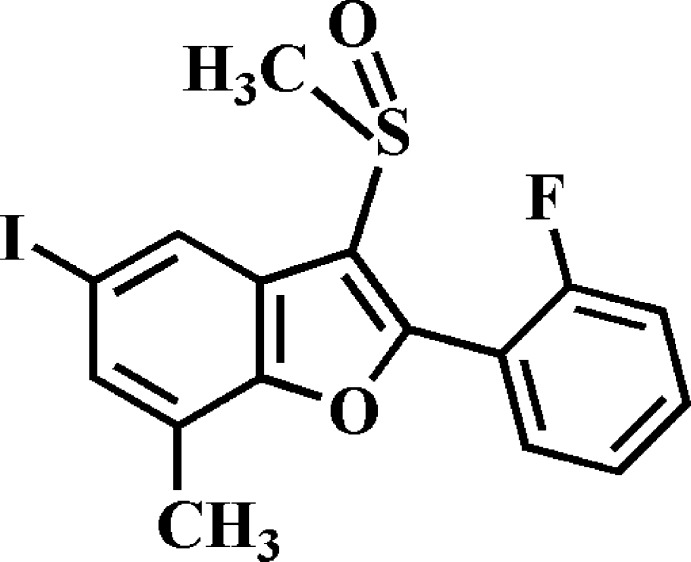


## Experimental   

### 

#### Crystal data  

C_16_H_12_FIO_2_S
*M* *_r_* = 414.22
Monoclinic, 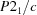

*a* = 7.9460 (2) Å
*b* = 24.1545 (7) Å
*c* = 7.9685 (2) Å
β = 100.997 (1)°
*V* = 1501.32 (7) Å^3^
*Z* = 4
Mo *K*α radiation
μ = 2.28 mm^−1^
*T* = 173 K
0.42 × 0.40 × 0.13 mm

#### Data collection  

Bruker SMART APEXII CCD diffractometer
Absorption correction: multi-scan (*SADABS*; Bruker, 2009 ▶) *T*_min_ = 0.541, *T*_max_ = 0.746
14688 measured reflections
3721 independent reflections
3465 reflections with *I* > 2σ(*I*)
*R*_int_ = 0.025

#### Refinement  

*R*[*F* ^2^ > 2σ(*F* ^2^)] = 0.026
*wR*(*F* ^2^) = 0.059
*S* = 1.12
3721 reflections
192 parameters
H-atom parameters constrained
Δρ_max_ = 0.47 e Å^−3^
Δρ_min_ = −0.56 e Å^−3^

### 

Data collection: *APEX2* (Bruker, 2009 ▶); cell refinement: *SAINT* (Bruker, 2009 ▶); data reduction: *SAINT*; program(s) used to solve structure: *SHELXS97* (Sheldrick, 2008 ▶); program(s) used to refine structure: *SHELXL97* (Sheldrick, 2008 ▶); molecular graphics: *ORTEP-3 for Windows* (Farrugia, 2012 ▶) and *DIAMOND* (Brandenburg, 1998 ▶); software used to prepare material for publication: *SHELXL97*.

## Supplementary Material

Crystal structure: contains datablock(s) I. DOI: 10.1107/S1600536814010459/hb7228sup1.cif

Structure factors: contains datablock(s) I. DOI: 10.1107/S1600536814010459/hb7228Isup2.hkl

Supporting information file. DOI: 10.1107/S1600536814010459/hb7228Isup3.cml

CCDC reference: 1001669

Additional supporting information:  crystallographic information; 3D view; checkCIF report

## Figures and Tables

**Table 1:** Hydrogen-bond geometry (Å, °)

*D*—H⋯*A*	*D*—H	H⋯*A*	*D*⋯*A*	*D*—H⋯*A*
C13—H13⋯O2^i^	0.95	2.49	3.355 (3)	151
C15—H15⋯O2^ii^	0.95	2.48	3.185 (3)	131

Symmetry codes: (i) 
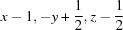
; (ii) 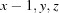.

**Fig. 1.**
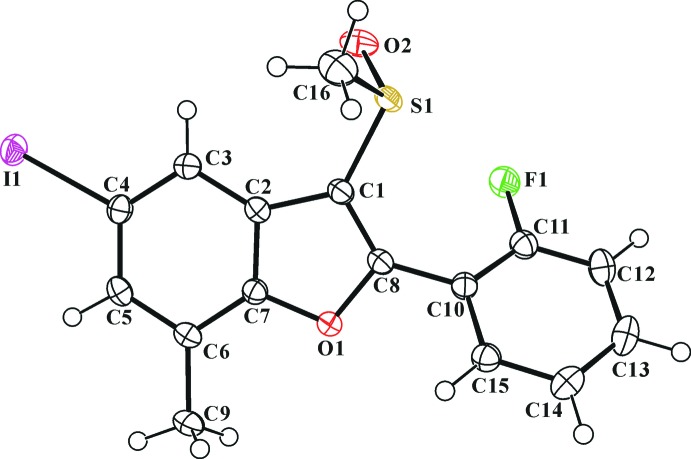
The molecular structure of the title molecule with displacement ellipsoids drawn at the 50% probability level.

**Fig. 2.**
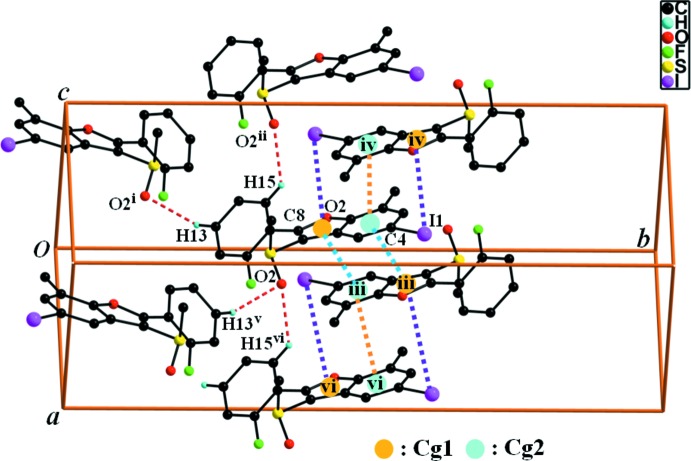
A view of the C–H···O, I···π and π···π interactions (dotted lines) in the crystal structure of the title compound. H atoms non-participating in hydrogen-bonding were omitted for clarity. [Symmetry codes: (i) x - 1, - y + 1/2, z - 1/2; (ii) x - 1, y, z; (iii)- x + 1, - y + 1, - z + 1; (iv) - x + 1, - y + 1, - z + 2; (v) x + 1, - y + 1/2, z + 1/2; (vi) x + 1, y, z.]

## supplementary crystallographic information

### 1. Comment

As a part of our ongoing study of 5-iodo-7-methyl-3-methylsulfinyl-1-benzofuran derivatives containing 4-fluorophenyl (Choi *et al.*, 2010), 3-fluorophenyl (Choi *et al.*, 2012) and 4-methylphenyl (Choi *et al.*, 2014) substituents in 2-position, we report here on the crystal structure of the title compound.

In the title molecule (Fig. 1), the benzofuran unit is essentially planar, with a mean deviation of 0.019 (2) Å from the least-squares plane defined by the nine constituent atoms. The 2-fluorophenyl ring is essentially planar, with a mean deviation of 0.012 (2) Å from the least-squares plane defined by the six constituent atoms. The dihedral angle formed by the benzofuran ring system and the 2-fluorophenyl ring is 39.78 (7)°. In the crystal structure (Fig. 2), molecules are linked *via* pairs of C4–I1···π contacts between the iodine atom and the furan ring of a neighbouring molecule with a C4–I1···Cg1^iv^ = 3.812 (2) Å (Cg1 is the C1/C2/C7/O1/C8 furan ring), and by a π···π interaction between the benzene rings of neighbouring molecules, with a Cg2···Cg2^iv^ distance of 3.821 (2) Å and an interplanar distance of 3.581 (2) Å resulting in a slippage of 1.333 (2) Å (Cg2 is the C2-C7 benzene ring), into inversion dimers. These dimers are further linked by π···π interactions between the furan and benzene rings of neighbouring molecules, with a Cg1···Cg2^iii^ distance of 3.668 (2) Å and an interplanar distance of 3.375 (2) Å resulting in a slippage of 1.437 (2) Å. The molecules stack along the a-axis direction. In addition, C–H···O hydrogen bonds (Table 1) are observed between inversion-related dimers.

### 2. Experimental

3-Chloroperoxybenzoic acid (77%, 224 mg, 1.0 mmol) was added in small portions to a stirred solution of 2-(2-fluorophenyl)-5-iodo-7-methyl-3-methylsulfanyl-1-benzofuran (358 mg, 0.9 mmol) in dichloromethane (40 mL) at 273 K. After being stirred at room temperature for 6h, the mixture was washed with saturated sodium bicarbonate solution and the organic layer was separated, dried over magnesium sulfate, filtered and concentrated at reduced pressure. The residue was purified by column chromatography (hexane-ethyl acetate, 1:1 *v*/*v*) to afford the title compound as a colorless solid [yield 72%, m.p. 469-470 K; *R*_f_ = 0.48 (hexane-ethyl acetate, 1:1 *v*/*v*)]. Colourless blocks were prepared by slow evaporation of a solution of the title compound in ethyl acetate at room temperature.

### 3. Refinement

All H atoms were positioned geometrically and refined using a riding model, with C–H = 0.95 Å for aryl, 0.99 Å for methyl H atoms. *U*_iso_ (H) = 1.2*U*_eq_ (C) for aryl and 1.5*U*_eq_ (C) for methyl H atoms. The methyl groups were allowed to rotate, but not to tip, to best fit the electron density.

### Crystal data

**Table:**

C_16_H_12_FIO_2_S	*F*(000) = 808
*M**_r_* = 414.22	*D*_x_ = 1.833 Mg m^−^^3^
Monoclinic, *P*2_1_/*c*	Melting point = 470–469 K
Hall symbol: -P 2ybc	Mo *K*α radiation, λ = 0.71073 Å
*a* = 7.9460 (2) Å	Cell parameters from 8240 reflections
*b* = 24.1545 (7) Å	θ = 2.6–28.3°
*c* = 7.9685 (2) Å	µ = 2.28 mm^−^^1^
β = 100.997 (1)°	*T* = 173 K
*V* = 1501.32 (7) Å^3^	Block, colourless
*Z* = 4	0.42 × 0.40 × 0.13 mm

### Data collection

**Table:**

Bruker SMART APEXII CCD diffractometer	3721 independent reflections
Radiation source: rotating anode	3465 reflections with *I* > 2σ(*I*)
Graphite multilayer monochromator	*R*_int_ = 0.025
Detector resolution: 10.0 pixels mm^-1^	θ_max_ = 28.4°, θ_min_ = 1.7°
φ and ω scans	*h* = −10→10
Absorption correction: multi-scan (*SADABS*; Bruker, 2009)	*k* = −32→27
*T*_min_ = 0.541, *T*_max_ = 0.746	*l* = −10→10
14688 measured reflections	

### Refinement

**Table:**

Refinement on *F*^2^	Primary atom site location: structure-invariant direct methods
Least-squares matrix: full	Secondary atom site location: difference Fourier map
*R*[*F*^2^ > 2σ(*F*^2^)] = 0.026	Hydrogen site location: difference Fourier map
*wR*(*F*^2^) = 0.059	H-atom parameters constrained
*S* = 1.12	*w* = 1/[σ^2^(*F*_o_^2^) + (0.0242*P*)^2^ + 1.1461*P*] where *P* = (*F*_o_^2^ + 2*F*_c_^2^)/3
3721 reflections	(Δ/σ)_max_ = 0.002
192 parameters	Δρ_max_ = 0.47 e Å^−^^3^
0 restraints	Δρ_min_ = −0.56 e Å^−^^3^

### Special details

**Table:**

Geometry. All esds (except the esd in the dihedral angle between two l.s. planes) are estimated using the full covariance matrix. The cell esds are taken into account individually in the estimation of esds in distances, angles and torsion angles; correlations between esds in cell parameters are only used when they are defined by crystal symmetry. An approximate (isotropic) treatment of cell esds is used for estimating esds involving l.s. planes.
Refinement. Refinement of F^2^ against ALL reflections. The weighted R-factor wR and goodness of fit S are based on F^2^, conventional R-factors R are based on F, with F set to zero for negative F^2^. The threshold expression of F^2^ > 2sigma(F^2^) is used only for calculating R-factors(gt) etc. and is not relevant to the choice of reflections for refinement. R-factors based on F^2^ are statistically about twice as large as those based on F, and R- factors based on ALL data will be even larger.

### Fractional atomic coordinates and isotropic or equivalent isotropic displacement parameters (Å^2^)

**Table:**

	*x*	*y*	*z*	*U*_iso_*/*U*_eq_	
I1	0.836495 (19)	0.586239 (6)	1.028521 (19)	0.02707 (6)	
S1	0.69187 (7)	0.33626 (2)	0.73135 (7)	0.02286 (12)	
F1	0.54937 (17)	0.31240 (6)	0.36409 (19)	0.0312 (3)	
O1	0.31221 (18)	0.43857 (6)	0.56014 (19)	0.0199 (3)	
O2	0.8687 (2)	0.35305 (8)	0.7161 (2)	0.0339 (4)	
C1	0.5544 (3)	0.39339 (9)	0.6766 (3)	0.0202 (4)	
C2	0.5700 (3)	0.44971 (9)	0.7398 (3)	0.0194 (4)	
C3	0.6952 (3)	0.48077 (9)	0.8473 (3)	0.0221 (4)	
H3	0.8019	0.4651	0.8998	0.026*	
C4	0.6553 (3)	0.53565 (9)	0.8730 (3)	0.0214 (4)	
C5	0.4976 (3)	0.55926 (9)	0.7989 (3)	0.0223 (4)	
H5	0.4764	0.5970	0.8215	0.027*	
C6	0.3717 (3)	0.52899 (9)	0.6932 (3)	0.0203 (4)	
C7	0.4166 (3)	0.47492 (9)	0.6656 (3)	0.0192 (4)	
C8	0.4009 (3)	0.38961 (9)	0.5680 (3)	0.0195 (4)	
C9	0.1982 (3)	0.55220 (10)	0.6186 (3)	0.0284 (5)	
H9A	0.1731	0.5456	0.4950	0.043*	
H9B	0.1973	0.5921	0.6408	0.043*	
H9C	0.1110	0.5340	0.6713	0.043*	
C10	0.3059 (3)	0.34517 (9)	0.4659 (3)	0.0205 (4)	
C11	0.3805 (3)	0.30761 (9)	0.3698 (3)	0.0240 (4)	
C12	0.2887 (3)	0.26551 (10)	0.2777 (3)	0.0311 (5)	
H12	0.3433	0.2403	0.2138	0.037*	
C13	0.1158 (3)	0.26053 (11)	0.2799 (3)	0.0336 (6)	
H13	0.0515	0.2310	0.2201	0.040*	
C14	0.0361 (3)	0.29845 (11)	0.3690 (3)	0.0298 (5)	
H14	−0.0836	0.2957	0.3669	0.036*	
C15	0.1300 (3)	0.34039 (10)	0.4610 (3)	0.0234 (4)	
H15	0.0741	0.3663	0.5215	0.028*	
C16	0.6912 (4)	0.33410 (12)	0.9561 (3)	0.0344 (6)	
H16A	0.7673	0.3044	1.0092	0.052*	
H16B	0.5745	0.3270	0.9740	0.052*	
H16C	0.7315	0.3696	1.0081	0.052*	

### Atomic displacement parameters (Å^2^)

**Table:**

	*U*^11^	*U*^22^	*U*^33^	*U*^12^	*U*^13^	*U*^23^
I1	0.02576 (9)	0.02477 (9)	0.02996 (9)	−0.00501 (5)	0.00348 (6)	−0.00525 (6)
S1	0.0252 (3)	0.0190 (3)	0.0238 (3)	0.0066 (2)	0.0033 (2)	−0.0005 (2)
F1	0.0262 (7)	0.0334 (8)	0.0364 (8)	0.0013 (6)	0.0118 (6)	−0.0074 (6)
O1	0.0192 (7)	0.0182 (8)	0.0213 (7)	0.0021 (6)	0.0018 (6)	−0.0012 (6)
O2	0.0231 (8)	0.0399 (10)	0.0395 (10)	0.0096 (7)	0.0082 (7)	0.0036 (8)
C1	0.0203 (10)	0.0188 (10)	0.0218 (10)	0.0031 (8)	0.0044 (8)	−0.0002 (8)
C2	0.0205 (10)	0.0174 (10)	0.0209 (10)	0.0010 (8)	0.0050 (8)	0.0005 (8)
C3	0.0200 (10)	0.0214 (11)	0.0242 (11)	0.0012 (8)	0.0028 (8)	−0.0002 (8)
C4	0.0227 (10)	0.0211 (11)	0.0209 (10)	−0.0032 (8)	0.0050 (8)	−0.0011 (8)
C5	0.0268 (11)	0.0162 (10)	0.0252 (11)	0.0006 (8)	0.0085 (9)	−0.0002 (8)
C6	0.0212 (10)	0.0192 (10)	0.0214 (10)	0.0036 (8)	0.0060 (8)	0.0025 (8)
C7	0.0189 (10)	0.0200 (11)	0.0192 (10)	0.0000 (8)	0.0049 (8)	−0.0010 (8)
C8	0.0209 (10)	0.0185 (10)	0.0193 (10)	0.0034 (8)	0.0050 (8)	0.0002 (8)
C9	0.0245 (11)	0.0232 (12)	0.0362 (13)	0.0075 (9)	0.0026 (10)	0.0011 (9)
C10	0.0236 (10)	0.0185 (11)	0.0183 (10)	0.0005 (8)	0.0011 (8)	0.0013 (8)
C11	0.0259 (11)	0.0233 (12)	0.0231 (11)	0.0014 (9)	0.0052 (9)	−0.0010 (8)
C12	0.0400 (14)	0.0257 (13)	0.0286 (12)	−0.0016 (10)	0.0089 (10)	−0.0084 (9)
C13	0.0404 (14)	0.0299 (13)	0.0289 (12)	−0.0116 (11)	0.0026 (10)	−0.0069 (10)
C14	0.0257 (11)	0.0348 (14)	0.0279 (12)	−0.0066 (10)	0.0024 (9)	0.0000 (10)
C15	0.0232 (10)	0.0235 (11)	0.0231 (11)	0.0006 (8)	0.0039 (8)	0.0006 (8)
C16	0.0433 (14)	0.0357 (14)	0.0246 (12)	0.0091 (11)	0.0077 (10)	0.0058 (10)

### Geometric parameters (Å, º)

**Table:**

I1—C4	2.102 (2)	C8—C10	1.466 (3)
S1—O2	1.4898 (18)	C9—H9A	0.9800
S1—C1	1.763 (2)	C9—H9B	0.9800
S1—C16	1.793 (2)	C9—H9C	0.9800
F1—C11	1.356 (3)	C10—C11	1.391 (3)
O1—C8	1.372 (3)	C10—C15	1.396 (3)
O1—C7	1.378 (2)	C11—C12	1.379 (3)
C1—C8	1.357 (3)	C12—C13	1.383 (4)
C1—C2	1.448 (3)	C12—H12	0.9500
C2—C7	1.389 (3)	C13—C14	1.384 (4)
C2—C3	1.400 (3)	C13—H13	0.9500
C3—C4	1.387 (3)	C14—C15	1.383 (3)
C3—H3	0.9500	C14—H14	0.9500
C4—C5	1.401 (3)	C15—H15	0.9500
C5—C6	1.387 (3)	C16—H16A	0.9800
C5—H5	0.9500	C16—H16B	0.9800
C6—C7	1.383 (3)	C16—H16C	0.9800
C6—C9	1.502 (3)		
			
O2—S1—C1	108.46 (10)	C6—C9—H9B	109.5
O2—S1—C16	105.79 (12)	H9A—C9—H9B	109.5
C1—S1—C16	98.65 (11)	C6—C9—H9C	109.5
C8—O1—C7	106.08 (16)	H9A—C9—H9C	109.5
C8—C1—C2	106.99 (18)	H9B—C9—H9C	109.5
C8—C1—S1	122.98 (17)	C11—C10—C15	117.4 (2)
C2—C1—S1	129.91 (16)	C11—C10—C8	123.7 (2)
C7—C2—C3	119.2 (2)	C15—C10—C8	118.9 (2)
C7—C2—C1	104.70 (18)	F1—C11—C12	118.1 (2)
C3—C2—C1	136.0 (2)	F1—C11—C10	119.4 (2)
C4—C3—C2	116.5 (2)	C12—C11—C10	122.4 (2)
C4—C3—H3	121.7	C11—C12—C13	118.9 (2)
C2—C3—H3	121.7	C11—C12—H12	120.5
C3—C4—C5	122.5 (2)	C13—C12—H12	120.5
C3—C4—I1	119.52 (16)	C12—C13—C14	120.1 (2)
C5—C4—I1	117.95 (16)	C12—C13—H13	119.9
C6—C5—C4	121.7 (2)	C14—C13—H13	119.9
C6—C5—H5	119.2	C15—C14—C13	120.2 (2)
C4—C5—H5	119.2	C15—C14—H14	119.9
C7—C6—C5	114.60 (19)	C13—C14—H14	119.9
C7—C6—C9	122.3 (2)	C14—C15—C10	120.8 (2)
C5—C6—C9	123.0 (2)	C14—C15—H15	119.6
O1—C7—C6	123.72 (19)	C10—C15—H15	119.6
O1—C7—C2	110.94 (18)	S1—C16—H16A	109.5
C6—C7—C2	125.3 (2)	S1—C16—H16B	109.5
C1—C8—O1	111.24 (18)	H16A—C16—H16B	109.5
C1—C8—C10	135.1 (2)	S1—C16—H16C	109.5
O1—C8—C10	113.59 (17)	H16A—C16—H16C	109.5
C6—C9—H9A	109.5	H16B—C16—H16C	109.5
			
O2—S1—C1—C8	132.80 (19)	C3—C2—C7—C6	−2.5 (3)
C16—S1—C1—C8	−117.3 (2)	C1—C2—C7—C6	178.2 (2)
O2—S1—C1—C2	−51.8 (2)	C2—C1—C8—O1	−2.2 (2)
C16—S1—C1—C2	58.1 (2)	S1—C1—C8—O1	174.07 (15)
C8—C1—C2—C7	2.1 (2)	C2—C1—C8—C10	−178.3 (2)
S1—C1—C2—C7	−173.83 (18)	S1—C1—C8—C10	−2.0 (4)
C8—C1—C2—C3	−177.0 (2)	C7—O1—C8—C1	1.4 (2)
S1—C1—C2—C3	7.0 (4)	C7—O1—C8—C10	178.36 (17)
C7—C2—C3—C4	0.4 (3)	C1—C8—C10—C11	−44.0 (4)
C1—C2—C3—C4	179.5 (2)	O1—C8—C10—C11	140.0 (2)
C2—C3—C4—C5	1.0 (3)	C1—C8—C10—C15	137.3 (3)
C2—C3—C4—I1	−178.86 (15)	O1—C8—C10—C15	−38.6 (3)
C3—C4—C5—C6	−0.6 (3)	C15—C10—C11—F1	176.76 (19)
I1—C4—C5—C6	179.31 (16)	C8—C10—C11—F1	−1.9 (3)
C4—C5—C6—C7	−1.3 (3)	C15—C10—C11—C12	−2.6 (3)
C4—C5—C6—C9	176.9 (2)	C8—C10—C11—C12	178.7 (2)
C8—O1—C7—C6	−179.5 (2)	F1—C11—C12—C13	−179.0 (2)
C8—O1—C7—C2	0.0 (2)	C10—C11—C12—C13	0.4 (4)
C5—C6—C7—O1	−177.70 (19)	C11—C12—C13—C14	2.0 (4)
C9—C6—C7—O1	4.1 (3)	C12—C13—C14—C15	−2.2 (4)
C5—C6—C7—C2	2.9 (3)	C13—C14—C15—C10	−0.1 (4)
C9—C6—C7—C2	−175.3 (2)	C11—C10—C15—C14	2.5 (3)
C3—C2—C7—O1	178.00 (18)	C8—C10—C15—C14	−178.8 (2)
C1—C2—C7—O1	−1.3 (2)		

### Hydrogen-bond geometry (Å, º)

**Table:**

*D*—H···*A*	*D*—H	H···*A*	*D*···*A*	*D*—H···*A*
C13—H13···O2^i^	0.95	2.49	3.355 (3)	151
C15—H15···O2^ii^	0.95	2.48	3.185 (3)	131

Symmetry codes: (i) *x*−1, −*y*+1/2, *z*−1/2; (ii) *x*−1, *y*, *z*.
